# The paradox of rapid and synchronized propagation of seasonal influenza ‘A’ outbreaks in contrast with COVID-19: a testable hypothesis

**DOI:** 10.1016/j.virusres.2025.199670

**Published:** 2025-11-26

**Authors:** Uri Gabbay, Doron Carmi

**Affiliations:** a Gray Faculty of Medicine and Health Sciences, Tel Aviv University, Israel; b Quality Unit, Rabin Medical Center, Israel; c Bat-Heffer Community Health Center, Clalit Health Services, Israel

**Keywords:** Biotic reservoirs, Bird migration, Influenza a virus, Outbreak propagation, Seasonal outbreaks, Viral transmission

## Abstract

Seasonal influenza A virus (SIAV) apparently exhibits a paradoxical pattern: despite a lower basic reproduction number (R₀) than SARS-CoV-2, it propagates across the Northern Hemisphere with remarkable speed and synchronicity. We propose a testable hypothesis, developed in two conceptual steps to explain this phenomenon.

First, we discuss what may explain the rapid, near-synchronous propagation of SIAV seasonal outbreak. We suggest that it may result from parallel seeding from multiple sources, rather than emerging from a singular origin, as observed with COVID-19. Second, we examined potential mechanisms for parallel seeding.

We propose a hypothesis-generating framework that, despite its limitations, offers a structured approach for integrating avian ecology with human epidemiology. The hypothesis is testable through genomic and metagenomic methods. Sequencing viruses from humans and migratory birds across regions may be evaluated to reveal identical viral lineages. The hypothesis may highlight the potential role of ecological reservoirs in global influenza propagation dynamics. If validated, this framework would advance understanding of influenza seasonality and may guide integrated surveillance strategies linking avian ecology with human epidemiology.

## 1 Introduction

Seasonal influenza A virus (SIAV) remains a major cause of global morbidity and mortality, with annual epidemics resulting in an estimated 290,000–650,000 respiratory deaths worldwide (Iuliano and Roguski, 2021, 2019). We wish to clarify that this hypothesis is solely related to seasonal influenza A and entirely unrelated to pandemic influenza. *“Seasonal influenza* A refers to annually recurring epidemics caused by human-adapted influenza A viruses. These viruses circulate globally, undergoing continuous antigenic drift. In contrast, *pandemic influenza* arises when a novel influenza A subtype, often originating from reassortment between avian, swine, or human strains, acquires efficient human-to-human transmissibility, leading to abrupt widespread infection in an immunologically naïve population. ( (Kilbourne, 2006; Taubenberger and Morens, 2006), Centers for Disease Control and Prevention (Centers for Disease Control and Prevention (CDC) 2023) 2023]

*Human influenza strains* are those adapted to efficient replication and transmission among humans, characterized by preferential binding to α2,6-linked sialic acid receptors. *Avian influenza strains* predominantly infect birds, binding to α2,3-linked receptors, and typically cause limited or non-sustained human transmission. (França et al., 2013; Nicholls et al., 2007; Stevens et al., 2006)

Although its basic reproduction number (R₀) is typically estimated between ∼1.2 and 1.8 (Lee et al., 2024), seasonal influenza outbreaks exhibit rapid and highly synchronized geographic propagation across Northern Hemisphere temperate regions. Not inferior to that of pandemic influenza spread (e.g., H1N1 2009) that exhibited moderate spread than SIAV epidemics (He et al., 2015).

The COVID-19 pandemic (SARS-CoV-2) provides a useful contrast for speed of propagation. While differences in surveillance sensitivity, population immunity, and diagnostic capacity must be considered, the estimated R₀ for COVID-19 ranged between 2 and 7 depending on the variant (Jenkins and Bible, 2021). Comparative epidemiologic parameters, including incubation period, infectious duration, and serial intervals, highlight that influenza’s transmissibility characteristics as exhibited by Table 1 seemingly do not comply with its higher propagation speed.

**Table 1:** Transmissibility characteristics of influenza seasonal outbreak compared with COVID-19.

Characteristic	Seasonal Influenza A (SIAV)	COVID-19 (SARS-CoV-2)	Key Feature
**Baseline Immunity in Population**	Variable, often 30–50 % due to prior exposure and vaccination (Iuliano and Roguski, 2019)	0 % at onset (novel virus) (Balsari, 2021)	Higher baseline immunity in SIAV
**Incubation Period**	∼1–4 days (Centers for Disease Control and Prevention. Seasonal Influenza (Flu) 2022)	∼2–14 days (Zhao and Huang, 2020)	Faster symptom onset in SIAV
**Duration of Illness**	∼5–7 days (Monto, 2002)	∼ 8–10 days after symptom onset. (Centers for Disease Control and Prevention 2021)	Shorter illness duration in SIAV
**Transmission Mode**	Spread mainly by droplets. fomite transmission is possible but not the major route; [(Centers for Disease Control and Prevention. Seasonal Influenza (Flu) 2022; Monto, 2002)]	Transmission Mode Droplets, fomites, and possibly airborne spread. (Van Doremalen et al., 2020)	More modes of transmission in COVID-19

SIAV evolution is characterized by continual antigenic drift driven by point mutations in hemagglutinin and neuraminidase, enabling immune escape and annual recurrence (Boni, 2008; Shao et al., 2017). The prevailing paradigm emphasizes viral persistence within human hosts between epidemics. Animal reservoirs, including avian populations seems noncontributing to SIAV outbreak (Shao et al., 2017).

### 1.1 What may explain the paradox

We hypothesize that this pattern may reflect that seasonal influenza outbreaks is initiated by parallel seeding in multiple remote locations, rather than emergence from a singular of origin, as occurs with pandemic influenza or SARS-CoV-2. If parallel seeding indeed occurs it provides a coherent explanation for the rapid global propagation of SIAV outbreak. We evaluated if the prevailing paradigm of humans as the sole seasonal reservoir can sufficiently explain initial parallel seeding mechanism.

**Can the Present Paradigm Human Reservoir Explain Possible Parallel Seeding?**

The prevailing paradigm assumes humans as the principal inter-epidemic reservoir with spread driven by human-to-human transmission modulated by environmental seasonality, international travel, and cyclical population immunity. Phylogeographic studies indicated that the human-only reservoir model does not fully explain global SIAV circulation (Lemey et al., 2022). Differences in detection and reporting provide only a partial explanation: COVID-19 initially circulated undetected, whereas seasonal influenza benefits from long-standing surveillance networks and clinical familiarity. Public health interventions during COVID-19, including travel restrictions, social distancing, and lockdowns, likely slowed perceived spread, but were generally enacted months after the initial outbreak, well beyond the typical nine-week duration of seasonal influenza peak global epidemics.

SIAV dissemination that induces multiple seedings is eventually still a single source, given humans are considered the sole reservoir.

Multiple independent emergences of identical influenza A subtypes require simultaneous emergence of identical viral subtypes in remote locations, along with the suppression of all other emerging subtypes. Traditional explanations invoking viral competition lacks empirical evidence.

### 1.2 Evolution of the hypothesis

The conceptual development of our hypothesis proceeded in two key steps. First, we suggest that SIAV rapid propagation may result from parallel seeding from multiple sources rather than emerging from a single origin. Second, we recognized that the prevailing paradigm assuming humans as the sole reservoir of influenza A cannot readily account for such parallel seeding. These observations imply that human-to-human transmission alone, even when influenced by environmental, behavioral, and public health factors, may not fully explain the observed speed and synchronicity of seasonal influenza propagation. Therefore, additional ecological or biological mechanisms may underline the process of parallel seeding.

### 1.3 The proposed hypothesis

We hypothesize that the dominant SIAV sub-strain emerges within a biotic reservoir during the summer breeding season. This reservoir is most probably within a northern body of water. Avian populations, including both domestic and migratory birds, may accumulate and maintain multiple viral lineages while cohabiting shared habitats. A certain SIAV subtype may thrive in the guts of migratory birds. During autumn migration, infected birds may disseminate this virus subtype along continental flyways, shedding the virus at stopover sites toward their end destinations. Proximity to human populations allows occasional, independent ecological introductions, generating initial near-simultaneous parallel infection seedings across geographically distant regions. Once introduced into humans, local transmission via conventional human-to-human contagiousness spreads, producing regional outbreaks. When these parallel introductions coincide with human susceptibility in temperate regions, rapidly propagating epidemics may occur.

**Fig. 1:**
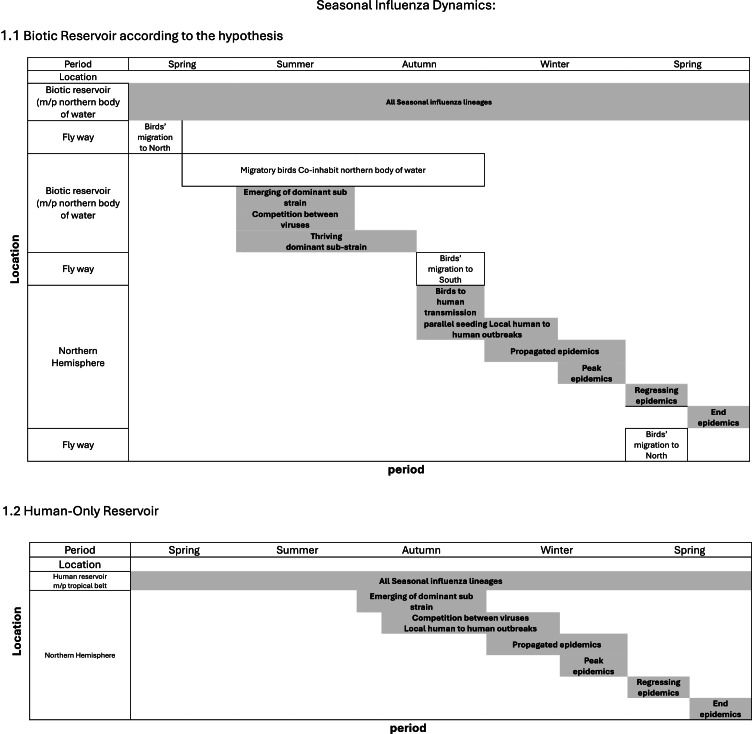
.

Fig. 1 demonstrated schematically the Seasonal Influenza Dynamics, according to the Biotic Reservoir according to the hypothesis and according to Human-Only Reservoir.

### 1.4 Supporting circumstantial evidence

Birds may carry human influenza subtypes in their intestines or secretions, retaining compatibility with α2,6-linked human sialic acid receptors, and thereby facilitating ecological transport (França et al., 2013; Nicholls et al., 2007; Stevens et al., 2006). These findings apparently support the biological feasibility of birds acting as ecological carriers of SIAV.

Unexpected reappearance of influenza A subtypes once believed to have vanished, such as the 1977 H1N1 re-emergence, highlights unresolved gaps in understanding influenza persistence and challenges the prevailing view that humans alone serve as the reservoir for seasonal influenza (JO. Wertheim, 2010).

Influenza A viruses are well-documented to circulate in wild waterfowl, particularly among *Anseriformes* and *Charadriiformes*, maintaining a genetically diverse viral gene pool (Gurley and Cummings, 2008; S Lindström and Olsen, 2007). Although direct evidence remains limited, migratory birds have been proposed as ecological reservoirs (Gurley and Cummings, 2008; Lindström and Olsen, 2007, 2007).

Many migratory waterfowl species congregate at high-density stopover sites and interact with diverse avian populations. These birds can shed influenza A viruses into aquatic environments, and viral RNA has been detected in lake and pond ice, demonstrating environmental persistence of influenza viruses associated with avian habitats (Rogers and McHugh, 2020), supporting the potential of SIAV biotic reservoir. Migratory bird flyways represent plausible pathways for long-distance dispersal of influenza A viruses among wild and domestic bird populations, potentially facilitating the geographic spread of viral subtypes across distant regions (Webster and Wetzel, 2007 Jul 15). The dispersal of influenza viruses by migratory wildfowl frequently occurs along major flyways, including those intersecting Southeast Asia, suggesting a potential ecological mechanism for virus transport that may precede human seasonal influenza outbreaks. (Gaidet et al., 2010). Moreover, Human-adapted influenza A subtypes, or highly similar reassortant variants, have been identified in poultry in advance of or concurrent with their detection in humans (Liu et al., 2014), suggesting potentially a bidirectional ecological exchange.

These observations provide indirect circumstantial, yet biologically plausible support for the hypothesis that migratory birds may contribute to the rapid, synchronized propagation of seasonal influenza.

### 1.5 What the hypothesis apparently explains

This hypothesis apparently may explain the rapid and nearly synchronous propagation of seasonal influenza across the Northern Hemisphere, despite its relatively moderate transmissibility (R₀). The proposed hypothesis may also provide a unified framework for several other enigmas related with SIAV. It may present a biologically plausible mechanism for the annual recurrence and pronounced seasonality of outbreaks not solely to climate conditions but also to birds’ migration. It may explain the dominance of a single viral sub-strain for each seasonal outbreak. Moreover, the hypothesis may account for the frequent initiation of outbreaks in Southeast Asia, consistent with the role of migratory bird pathways. It may also explain the re-emergence of sub-strains after prolonged apparent absence, as seen in historical events such as the 1977 H1N1 reappearance (JO. Wertheim, 2010; Zakstelskaja et al., 1978). This demonstrates that SIAV can reappear after decades with minimal antigenic change, consistent with the possibility of long-term maintenance and subsequent reseeding. If cold temperatures and low humidity were the sole determinants of seasonal influenza spread, we would expect epidemics to occur earlier at higher latitudes. However, observed epidemic timing varies across regions, and in some cases, lower-latitude locations in the Northern Hemisphere experience influenza peaks earlier than higher-latitude areas, indicating that additional factors influence outbreak initiation (Zanobini et al., 2022).

While these epidemiological patterns are well documented, the role of avian-mediated ecological transport remains hypothetical. Nevertheless, this framework integrates ecological, evolutionary, and epidemiological perspectives to generate explicitly testable predictions, offering a conceptual foundation for empirical studies that can evaluate the contribution of non-human reservoirs to human influenza seasonality, global synchronization, and subtype dynamics.

### 1.6 Testable measures and empirical validation

The proposed hypothesis is directly amenable to empirical testing through genomic, ecological, and epidemiologic approaches. Several measurable predictions can be evaluated:

1. Sequencing influenza A viruses from migratory birds and human populations along major flyways (e.g., Siberia-Southeast Asia) expected to reveal high phylogenetic similarity between early-season human isolates and avian strains sampled from overlapping migratory pathways.
2. Outbreak onset timing across regions expected to correlate with seasonal migration schedules rather than with latitude or local temperature alone (Temporal Spatial Correlation).
3. Metagenomic detection of human-adapted influenza subtypes in bird fecal or environmental samples during inter-epidemic periods would support the hypothesis of biotic persistence.
4. Annual dominance of a single sub-strain may correspond to avian reservoir bottlenecking or selective reseeding events, observable through longitudinal genomic surveillance.
5. Outbreak initiation may align more closely with migration-linked ecological introductions than with the lowest temperatures or highest humidity periods predicted by traditional seasonality models.

Collectively, these measures provide a practical roadmap for validating or refuting the proposed ecological reservoir hypothesis. Coordinated international surveillance, integrating viral genomics with ornithological tracking, could empirically determine whether migratory birds contribute to parallel seeding and synchronization of seasonal influenza outbreaks.

## 2 Limitations

Several limitations should be acknowledged. First, direct evidence supporting the parallel seeding of SIAV via migratory birds is currently lacking. The supporting observations including temporal alignment with migratory patterns, spatial synchrony across distant human populations, and partial phylogenetic overlap remain circumstantial and do not establish causality. The proposed hypothesis assumes that the explanation for the rapid propagation of SIAV is indeed parallel seeding. However, it does not exclude others, yet undiscovered alternative explanations, whether through parallel seeding or other mechanisms.

Second, while comparisons with COVID-19 provide an instructive contrast in propagation dynamics, the ecological and epidemiological contexts of the two viruses differ substantially. Accordingly, such comparisons should be regarded as heuristic rather than definitive.

Third, empirical validation of the proposed mechanism poses significant logistical and methodological challenges. It would require coordinated, longitudinal surveillance of both migratory birds and human populations across multiple continents, combined with viral genomic and phylogenetic analyses to differentiate avian-mediated introductions from early undetected human transmission chains. Current global surveillance infrastructures remain limited, and environmental or host-related factors may further complicate detection efforts.

Experimental studies suggest that the presence of both α2,3 and α2,6-linked sialic acid receptors can facilitate avian influenza A virus binding and entry, implying that mixed receptor environments may support cross-species viral interactions (Liu et al., 2022). These may suggest that only a small, specific subset of the vast migratory bird population may facilitate ecological transmission. This presents a significant statistical challenge for detection and analysis.

## 3 Conclusion and implications

SIAV continues to challenge prediction and control due to its rapid and nearly synchronous propagation across the Northern Hemisphere. We propose a hypothesis-generating framework that, despite its limitations, offers a structured approach for integrating avian ecology with human epidemiology. By explicitly acknowledging uncertainty and outlining feasible research directions, this hypothesis aims to stimulate empirical investigation rather than assert definitive proof. This ecological mechanism may enable parallel seeding across multiple human populations, preceding sustained human-to-human transmission, and provides a potential explanation for the paradoxical propagation speed of SIAV outbreaks.

The framework also offers conceptual resolution for several longstanding enigmas of influenza ecology, including annual recurrence, seasonal timing, dominance of a single sub-strain, suppression of competing strains during SIAV outbreaks, frequent initiation in Southeast Asia, and the reappearance of sub-strains after years of apparent absence.

Integrating avian surveillance with human epidemiology could enhance early outbreak detection, improve vaccine strain selection, and identify transmission hotspots. By incorporating biotic reservoirs into influenza ecology, this model reconciles observed outbreak dynamics, informs integrated surveillance strategies, and strengthens preparedness for seasonal influenza, while providing a foundation for empirical validation through genomic, ecological, and epidemiologic studies.

While bird-mediated transmission has been proposed previously, its potential role as an initial parallel-seeding mechanism that accelerates global outbreak propagation represents a novel, testable hypothesis. Although this framework remains hypothetical, it may help resolve one of the most persistent paradoxes in infectious disease dynamics.

## CRediT authorship contribution statement

**Uri Gabbay:** Writing – original draft, Investigation, Conceptualization. **Doron Carmi:** Writing – review & editing, Investigation, Conceptualization.

## Declaration of competing interest

The authors declare no conflict of interests

None of the authors received any financial support related to this manuscript

## Data Availability

this is a hypothesis paper based on published manuscript.
